# Parameter Optimization Analysis of Prolonged Analgesia Effect of tDCS on Neuropathic Pain Rats

**DOI:** 10.3389/fnbeh.2017.00115

**Published:** 2017-06-13

**Authors:** Hui-Zhong Wen, Shi-Hao Gao, Yan-Dong Zhao, Wen-Juan He, Xue-Long Tian, Huai-Zhen Ruan

**Affiliations:** ^1^Department of Neurobiology, College of Basic Medical Science, Chongqing Key Laboratory of Neurobiology, Third Military Medical UniversityChongqing, China; ^2^Department of Pathophysiology and High Altitudepathology, College of High Altitude Military Medicine, Third Military Medical UniversityChongqing, China; ^3^Bioengineering College, Chongqing UniversityChongqing, China

**Keywords:** transcranial direct current stimulation, tDCS, chronic constriction injury, neuropathic pain, intervention time, parameter optimization

## Abstract

**Background**: Transcranial direct current stimulation (tDCS) is widely used to treat human nerve disorders and neuropathic pain by modulating the excitability of cortex. The effectiveness of tDCS is influenced by its stimulation parameters, but there have been no systematic studies to help guide the selection of different parameters.

**Objective**: This study aims to assess the effects of tDCS of primary motor cortex (M1) on chronic neuropathic pain in rats and to test for the optimal parameter combinations for analgesia.

**Methods**: Using the chronic neuropathic pain models of chronic constriction injury (CCI), we measured pain thresholds before and after anodal-tDCS (A-tDCS) using different parameter conditions, including stimulation intensity, stimulation time, intervention time and electrode located (ipsilateral or contralateral M1 of the ligated paw on male/female CCI models).

**Results**: Following the application of A-tDCS over M1, we observed that the antinociceptive effects were depended on different parameters. First, we found that repetitive A-tDCS had a longer analgesic effect than single stimulus, and both ipsilateral-tDCS (ip-tDCS) and contralateral-tDCS (con-tDCS) produce a long-lasting analgesic effect on neuropathic pain. Second, the antinociceptive effects were intensity-dependent and time-dependent, high intensities worked better than low intensities and long stimulus durations worked better than short stimulus durations. Third, timing of the intervention after injury affected the stimulation outcome, early use of tDCS was an effective method to prevent the development of pain, and more frequent intervention induced more analgesia in CCI rats, finally, similar antinociceptive effects of con- and ip-tDCS were observed in both sexes of CCI rats.

**Conclusion**: Optimized protocols of tDCS for treating antinociceptive effects were developed. These findings should be taken into consideration when using tDCS to produce analgesic effects in clinical applications.

## Introduction

Chronic neuropathic pain is a common and severely disabling state that typically develops when peripheral nerves are damaged due to surgery, bone compression in cancer, diabetes or infection (Dworkin et al., 2013). Recent studies indicate increased activation of descending modulatory circuits (both descending facilitation and inhibition) in chronic neuropathic pain syndrome, and these circuit changes reflect long-lasting changes in synaptic efficacy (Zhuo, 2008, 2013). An increasing number of investigators hold the view that non-invasive brain stimulation techniques can be used to treat chronic neuropathic pain (O’Connell et al., 2011; Volz et al., 2012). The aim of brain stimulation in managing of pain is to reduce pain symptoms by altering activity in brain areas that are involved in processing painful stimuli (Nguyen et al., 2011; Mylius et al., 2012; Woods et al., 2016).

Repetitive transcranial magnetic stimulation (rTMS) and transcranial direct current stimulation (tDCS) are two typical and common techniques of non-invasive brain stimulation techniques (Woods et al., 2016). Relative to invasive stimulation, non-invasive stimulation requires no surgical procedure and is therefore easier and safer to administer. However, there are several advantages of tDCS over rTMS, such as lower cost, increased portability, and more convincing sham conditions (Zaghi et al., 2009). TDCS is considered as a neuromodulatory intervention for the brain (Nitsche et al., 2008) which modulates the membrane potential dependently by type of electrode’s application. Anode-tDCS (A-tDCS) is able to facilitate the depolarization of neurons and increase the cortical excitability. However, cathode-tDCS (C-tDCS) hyperpolarizes the resting membrane potential and reduces the cortical excitability (Nitsche et al., 2008; Mylius et al., 2012). Numerous clinical studies have concluded that A-tDCS is an effective method for pain modulations, which is helpful at reducing both fibromyalgia and spinal cord injury related-pain (Fregni et al., 2006; Roizenblatt et al., 2007; Soler et al., 2010). There are also studies showing effects of A-tDCS in inflammatory and neuropathic pain in animals (Laste et al., 2012; Spezia Adachi et al., 2012; Cioato et al., 2016; Filho et al., 2016).

Sensory-motor cortex has been reported to decrease pain sensation and to increase pain threshold (Xie et al., 2009; Ossipov et al., 2010). As a result, the primary motor cortex (M1) is regarded as the location for stimulation electrode placement in the vast majority of trials in patients (Ferrucci et al., 2015; Woods et al., 2016), but there was little research testing the location of the stimulation electrode in animal research, the stimulation electrodes of those studies were placed on the middle of the scalp of rat rather than on a particular location (Ferrucci et al., 2015; Woods et al., 2016). There are three location modes for electrodes as related to the region of pain: contralateral M1, bilateral M1 and ipsilateral M1 to the injured region. A-tDCS delivered to contralateral M1 and bilateral M1 have been reported to have an antinociceptive effect in a number of patients with chronic pain (Ngernyam, 2014; O’Neill et al., 2015; Woods et al., 2016), whereas few studies of tDCS of ipsilateral M1 have been reported.

Beside the location of the stimulation electrode, physical parameters and practical applications of tDCS are important factors in treating neuropathic pain in animal and clinical experiments (Nitsche et al., 2008; Nitsche and Paulus, 2011; Stagg and Nitsche, 2011). Therefore, tDCS protocols should state current intensity, electrode size, stimulation duration and other parameters to aid in assessing comparability among studies (Nitsche et al., 2008). Originally, tDCS was believed to follow simple rules: the more electric charge flowing through the electrode, the stronger the analgesic effect would be (Nitsche et al., 2008; Nitsche and Paulus, 2011). However, the safety and side effects of tDCS on participants should be precisely calculated, because high current density can cause tissue damage (Nitsche et al., 2008; Liebetanz et al., 2009). Another important parameter of tDCS is the intervention time, which involves the time when stimulation is administered in relation to the course of pain processing and how many times tDCS is delivered. According to previous findings, repetitive stimulation has been proven to enhance efficacy and prolong after-effects of tDCS during specific time intervals (Nitsche et al., 2008). In addition, because the course of neuropathic pain is fairly long and easy to be targeted repeatedly (Dworkin et al., 2013), it will be significant if the patient can be treated when the pain is onset with negligible side effects.

Therefore, a combination of tDCS stimulation parameters including current intensity as well as stimulation time, interval and position need to be studied systematically in clinical and animal experiments. However, due to the limitation of choosing suitable parameters in clinical studies, optimal protocol characteristics have not been explored systematically (Ngernyam, 2014). In this article, we conducted studies to explore the optimal physical parameters and practical applications of A-tDCS in treating chronic neuropathic pain in both male and female rats.

## Materials and Methods

### Experimental Animals

Experiments were carried out on adult Sprague–Dawley rats (8–10 weeks old weighing 220–250 g), which were purchased from the Center of Laboratory Animal, Third Military Medical University, Chongqing, China. The SD rats were housed in plastic cages with soft bedding under controlled temperature settings (24 ± 1°C), humidity (60 ± 5%) and a 12-h light/dark cycle. The study, animal care and handling procedures were in strict accordance with the recommendations of International Association for the Study of Pain’s ethical guidelines (Zimmermann, 1983), and the protocol was also approved by the Ethical Committee for Animal Research of Third Military Medical University.

### Animal Model of Neuropathic Pain

A typical neuropathic pain model was established through chronic constriction injury (CCI) of the sciatic nerve (Bennett and Xie, 1988).The right sciatic nerve of rats was tied with four 4-0 chromic gut ligatures 1 mm apart under 4% chloral hydrate-anesthesia (10 ml/kg, i.p.). The sutures were not tied so tight that blood flow was affected. The overlying muscle was sutured and the skin wound was sealed with topical antibiotics. Rats with right sciatic nerve exposed without chromic gut ligature served as sham CCI controls.

### Transcranial Direct Current Stimulation

**Figure 1:**
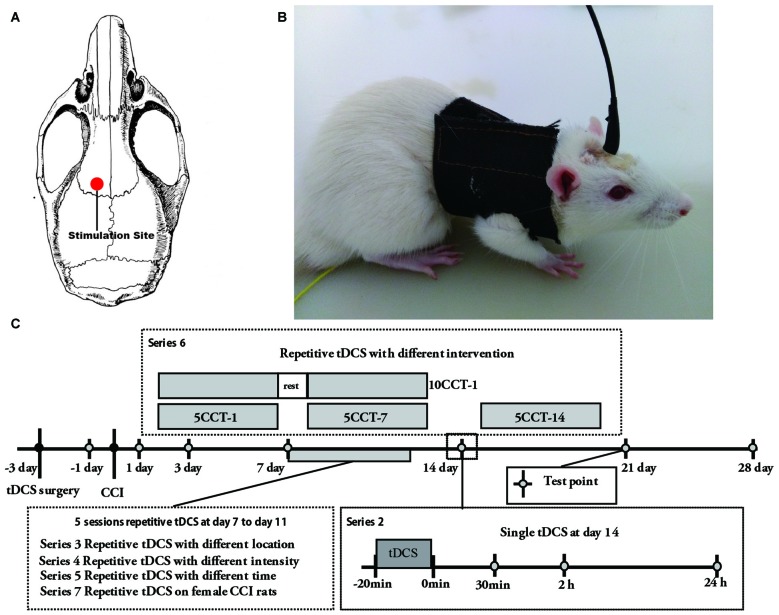
**(A)** Schematic diagram displaying the location of the stimulation electrode in the rat brain model. **(B)** Electrode configurations in rat. Two electrodes were applied during transcranial direct current stimulation (tDCS). The stimulation electrode was fixed onto the skull and a reference electrode was attached onto the thorax. **(C)** Schematic diagram of the experiment protocol schedule. 5CCT-1/5CCT-7/5CCT-14 means five times daily contralateral-tDCS (con-tDCS) started on day 1, 7, or 14 following chronic constriction injury (CCI), respectively; 10CCT-1 means 10 times con-tDCS at 2 weeks. Test point was the day to test the mechanical allodynia and thermal hyperalgesia on the hind paw of CCI.

We improved the previously implanted electrode protocol by optimizing the internal structural stability and by decreasing the contact impedance of the electrode (Liebetanz et al., 2006; Yu et al., 2015). Three days before CCI or sham CCI surgery, a saline soaked sponge was placed at the end of a plastic tube (inner diameter: 2 mm; length: 1 cm; Figures 1A,B). A copper wire was inserted into the sponge and held in place inside the tube with polyacrylate adhesive. Next, the tube was fixed with glass ionomer cement onto the cranium over M1 as a stimulation electrode using a stereotaxic apparatus; ipsilateral tDCS (ip-tDCS) refers to a stimulation electrode being fixed overlying the ipsilateral M1 to the ligated hind paw, and contralateral tDCS (con-tDCS) refers to stimulation electrode fixed overlying the contralateral M1 to the ligated hind paw (Figure 1A). A far larger conventional sponge electrode (10 cm^2^) was placed on the ventral thorax with a corset and served as a reference electrode (Figure 1B).

As described below, constant current was applied via the stimulation electrode as A-tDCS at the schedule times (1st, 7th or 14th days after CCI; Figure 1C). For sham tDCS, the stimulation electrode was placed in the same positions as for real stimulation, but stimulation duration of 10 s was used as described above (Yu et al., 2015). Meanwhile, at the beginning and end of tDCS, current was ramped up and down for 10 s to prevent damage to the brain tissues by suddenly changing current (Bindman et al., 1964; Yu et al., 2015). Saline was injected continuously into the sponge through the hole that we left on the top of the tube during the DC stimulation in order to reduce contact impedance when stimulating (Yu et al., 2015). Notably, animals were not anesthetized during tDCS or sham tDCS.

### Experimental Protocols

The experiment was composed by seven series (Figure 1C, Supplementary Table S1):

- **Series 1**: changes of pain thresholds after CCI. Thirty-three male rats were randomly divided into three groups: control (CT), sham CCI (SC) and CCI. Pain thresholds were tested 1 day before the CCI surgery and at days 1, 3, 7, 10, 14, 21 and 28 after CCI surgery.
- **Series 2**: changes of pain thresholds after single A-tDCS on CCI rats. On day 14 after CCI surgery, contralateral or ipsilateral A-tDCS was delivered in a single session of 20 min with stimulation current intensity of 100/200 μA. Male rats were divided into 12 groups: SC, sham CCI + sham 200 μA ip-tDCS (SCSIT), sham CCI + 200 μA sham con-tDCS (SCSCT), sham CCI + 200 μA ip-tDCS (SCIT), sham CCI + 200 μA con-tDCS (SCCT), CCI, CCI + sham 200 μA ip-tDCS (CSIT), CCI + sham 200 μA con-tDCS (CSCT), CCI + 100 μA ip-tDCS (CIT100), CCI + 200 μA ip-tDCS (CIT200), CCI + 100 μA con-tDCS (CCT100) and CCI + 200 μA con-tDCS (CCT200; *n* = 11 per group, of which were observed before, and 30 min, 2 h and 24 h after A-tDCS or sham A-tDCS).
- **Series 3**: changes of pain thresholds after processed with repetitive ip-tDCS and con-tDCS on CCI rats. Daily A-tDCS (20 min/200 μA) was applied over five sessions over the contralateral or ipsilateral M1 from the 7th day after CCI. In this series, male rats were divided into four groups: SC, CCI, CIT and CCT.
- **Series 4**: observation of pain thresholds changes after repetitive contralateral A-tDCS with different intensities. Five times daily A-tDCS with different intensities was applied for 20 min on the contralateral M1 from the 7th day after CCI. In this series, male rats were divided into six groups: SC, CCI, CCI + 15 μA con-tDCS (CCT15), CCI + 50 μA con-tDCS (CCT50), CCI + 100 μA con-tDCS (CCT100), and CCI + 200 μA con-tDCS (CCT200).
- **Series 5**: observation of pain thresholds changes after repetitive contralateral A-tDCS with different times. Daily 200 μA A-tDCS was applied on five sessions over the contralateral M1 from the 7th day after CCI. In this series, male rats were divided into six groups: SC, CCI, CCI + 5 min con-tDCS (CCT200-5), CCI + 10 min con-tDCS (CCT200-10), CCI + 20 min con-tDCS (CCT200-20), and CCI + 30 min con-tDCS (CCT200-30).
- **Series 6**: changes in the pain thresholds after A-tDCS on CCI model with different intervention time and sessions. In this series, CCI rats were given with five sessions of repetitive contralateral A-tDCS (20 min/200 μA) on the 1st day (5CCI-1), 7th day (5CCI-7) and 14th day (5CCI-14) after CCI surgery. Other rats were given 10 sessions of repetitive tDCS (20 min/200 μA) starting on the 1st day (10CCT-1) after CCI surgery. Male rats were divided into six groups: SC, CCI, 5CCT-1, 5CCT-7, 5CCT-14, and 10CCT-1.
- **Series 7**: changes in the pain thresholds after repetitive A-tDCS on female rats of the CCI model. Fifty-five female rats were divided into five groups: CT, SC, CCI, CCT and CIT, and daily A-tDCS (20 min/200 μA) was applied on five sessions over the contralateral or ipsilateral M1 from the 7th day after CCI.
- **Series 2–7**: pain thresholds were tested 1 day before CCI surgery and on days 1, 3, 7, 10, 14, 21 and 28 after CCI surgery (*n* = 11, per group).

### Radiant Heat Test

The radiant heat test was carried out to estimate the thermal withdrawal latency (TWL; Hargreaves et al., 1988). After an adaptation period of 30 min, the rats were placed into the test cage with a glass plate under which a light was located; 52 ± 0.2°C radiant heat was applied to the plantar surface of the right hind-paw. The latency period was recorded in response to the thermal hyperalgesia by lifting hind-paw licking, flicking or commences jumping. To avoid tissue injury, the cut-off limit was set at 60 s (Hargreaves et al., 1988). Each hind-paw was measured for three times alternately at a 5 min interval. The mean was recorded as TWL.

### Von Frey Filaments Test

The von Frey filaments test using an up-down method was performed to estimate the 50% system mechanical withdrawal threshold (MWT) with bending forces at a range of 0.3–20.3 g von Frey hairs (vFh; Chaplan et al., 1994). Each rat was placed inside a transparent acrylic cage (18 cm × 12 cm × 12 cm) with wire mesh floor with 60 min of acclimatization. The test was initiated with 4.10 g vFh. The filament was applied to the ventral surface of each right hind-paw for 4–6 s, hind paw withdrawal was considered as a positive response. When a positive result was noted, then the filament was decremented by one step size. If a negative result occurs, the filament was increased. The test continues until four measurements have been made after the first change in direction.

### Statistical Analysis

**Figure 2:**
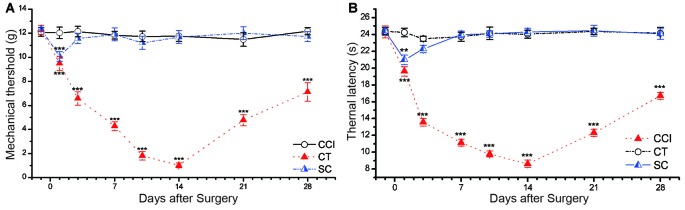
CCI induced significant mechanical allodynia and thermal hyperalgesia in rat. **(A)** Mechanical withdrawal threshold (MWT). **(B)** Thermal withdrawal latency (TWL). Control (CT); sham CCI (SC). All behavior tests were examined 1 day before the CCI surgery and on days 1, 3, 7, 10, 14, 21 and 28 after CCI surgery. Statistical significance was analyzed by two-way analysis of variance (ANOVA) followed by Tukey’s *post hoc* test. The data are shown as mean ± SEM; **p < 0.01, ***p < 0.001 vs. CT group.

Analyses were done with the SPSS software package (version 19). All data are expressed as the mean ± SD. The pain thresholds were evaluated by one way (Figure 2) or two-way (except Figure 2) repeated measure analysis of variance (ANOVA), when significant differences were observed, a *post hoc* test was made via Tukey’s test. In all cases, *p* < 0.05 was considered to be statistically significant.

## Results

### Changes of Pain Thresholds in CCI Rats

Significant mechanical allodynia and thermal hyperalgesia were elicited in surgical hind paw CCI rats (Figure 2). Two-way repeated measures ANOVA showed a significant main effect of group on the MWT (main effect of group *F*_(2,30)_ = 663.718, *p* = 0.000) and TWL (*F*_(2,30)_ = 828.857, *p* = 0.000). The values of MWT and TWL were decreased starting 1 day after CCI surgery, and the most severe stage appeared around day 14 (MWT: 1.03 ± 0.21 g, TWL: 8.61 ± 0.44 s) and then began to recover on the following day (Figure 2). This trend was in line with our previous studies (Xiao et al., 2010; Ou et al., 2011; He et al., 2012). There was no significant difference in the thermal latency and mechanical threshold between control and sham CCI groups (*p* = 0.259 of TWL, *p* = 0.128 of MWT).

### Effects of Single tDCS Treatment Disappeared within 24 h after Stimulation

We chose the 14th day after CCI to test the duration of antinociceptive effects after one time tDCS.

**Figure 3:**
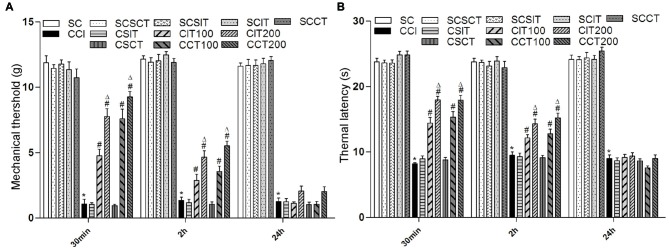
Effects of single tDCS on mechanical allodynia and thermal hyperalgesia. All behavior tests were examined before, and then 30 min, 2 h and 24 h after tDCS. Administration of tDCS started on day 14 after CCI surgery. Experimental groups include, sham CCI (SC); CCI; sham CCI + sham 200 μA ip-tDCS (SCSIT); sham CCI + sham 200 μA con-tDCS (SCSCT); sham CCI + 200 μA ip-tDCS (SCIT); sham CCI + 200 μA con-tDCS (SCCT); CCI + sham 200 μA ip-tDCS (CSIT); CCI + sham 200 μA con-tDCS (CSCT); CCI + 100 μA ip-tDCS (CIT100); CCI + 200 μA ip-tDCS (CIT200); CCI + 100 μA con-tDCS (CCT100); CCI + 200 μA con-tDCS (CCT200). **(A)** MWT. **(B)** TWL. Group analysis was performed by one-way ANOVA followed by Tukey’s test. In all three test phases, there were no difference between SC and SCSIT, SCSCT, SCIT, SCCT groups, and there was also no difference between CCI and CSCT, CSIT groups. However, there were significant differences between SC, SCSIT, SCSCT, SCIT, SCCT and CCI groups (all *p < 0.05 vs. CCI group). 30 min: the CIT and CCT groups (both 100 μA and 200 μA) differ from the CCI, CSIT and CSCT groups; 2 h: the CIT and CCT groups (both 100 μA and 200 μA) differ from the CCI, CSIT and CSCT groups; 24 h: there is no significant difference from CCI, CSIT, CSST, CIT and CCT groups (^#^*p* < 0.05 compared with CCI group, and ^Δ^*p* < 0.05 compared with CIT100 or CCT100 group).

No difference was found in TWL and MWT among SC, SCSIT, SCSCT, SCIT and SCCT groups at all test points (one-way ANOVA, all *p* > 0.05). Sham tDCS (both SCSCT and SCSIT) had no impact on behaviors of Sham CCI rats, and the tDCS (both SCIT and SCCT) also did not have impact on behaviors of sham CCI rats (one-way ANOVA, all *p* > 0.05; Figure 3).

Compared with CCI, single con-tDCS (both CCT100 and CCT200) sharply increased TWL and MWT after one session of A-tDCS that ended 30 min before behavioral tests (one-way ANOVA, all *p* = 0.000). The effects were gradually diminished in the following 2 h (one-way ANOVA, TWL: *p* = 0.000 for CCT100, *p* = 0.000 for CCT200; MWT: *p* = 0.001 for CCT100, *p* = 0.000 for CCT200 vs. CCI). After 24 h, the antinociceptive effects were almost completely gone and there were no differences compared with the CCI group (one-way ANOVA, all *p* > 0.05). Moreover, we also observed a better recovery of pain in CCT200 than CCT100 (one-way ANOVA, TWL: *p* = 0.044 for 30 min, *p* = 0.004 for 2 h, *p* = 0.490 for 24 h; MWT: *p* = 0.037 for 30 min, *p* = 0.009 for 2 h, *p* = 0.523 for 24 h).

Similarly, single ip-tDCS (CIT100 and CIT200) also showed analgesia effects on CCI rats (one-way ANOVA, 30 min: all *p* = 0.000 vs. CCI). However, compared to the effects observed with con-tDCS, the antinociceptive effects decreased in the following 2 h (one-way ANOVA, TWL: *p* = 0.005 for CIT100 and *p* = 0.000 for CIT200; MWT: *p* = 0.010 for CIT100 and *p* = 0.000 for CIT200) and disappeared after 24 h with ip-tDCS (one-way ANOVA, all *p* > 0.05; Figure 3). We also observed increased pain recovery in the CIT200 group compared to the CIT100 group (one-way ANOVA, TWL: *p* = 0.000 for 30 min, *p* = 0.006 for 2 h, *p* = 1.000 for 24 h; MWT: *p* = 0.001 for 30 min, *p* = 0.028 for 2 h, *p* = 0.582 for 24 h; Figure 3).

However, we did not observed changes in pain thresholds of sham tDCS (both CSIT and CSCT) in CCI rats (one-way ANOVA, all *p* > 0.05; Figure 3).

### Both ip- and con-Repetitive tDCS Had Long-Term Antinociceptive Effects in CCI Rats

**Figure 4:**
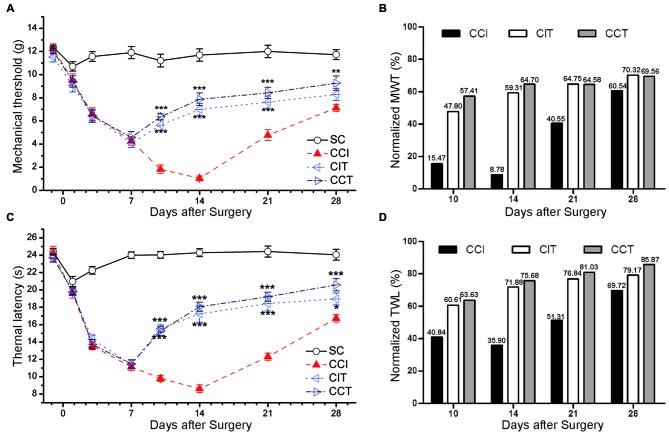
Effects of repetitive tDCS on mechanical allodynia and thermal hyperalgesia with different electrode locations. **(A)** MWT. **(B)** Normalized of MWT. **(C)** TWL. **(D)** Normalized of TWL. Five sessions daily of tDCS (200 μA, 20 min) were administered starting on day 7 after CCI surgery. SC (sham CCI); CCI + ip-tDCS (CIT); CCI + con-tDCS (CCT). CIT and CCT treatments significantly increased the values of TWL and MWT. All behavior tests were tested 1 day before the CCI surgery and on days 1, 3, 7, 10, 14, 21 and 28 after CCI surgery. Statistical significance was analyzed by two-way ANOVA followed by Tukey’s *post hoc* test. *p < 0.05, **p < 0.01, ***p < 0.001 vs. CCI group.

Repetitive A-tDCS had long-term antinociceptive effects, significant increases in TWL and MWT were observed not only during the stimulation process, but also 1 or more weeks following A-tDCS (Figures 4A,C). Five sessions of repetitive A-tDCS had similar antinociceptive effects in both CIT and CCT groups; similar effects were observed in surgical hind paw of CCI rats (two-way repeated measures ANOVA: TWL: *F*_(3,40)_ = 480.888, *p* = 0.000; MWT: *F*_(3,40)_ = 150.201, *p* = 0.000. Tukey’s test of groups: all *p* = 0.000 vs. CCI). Moreover, the values of TWL and MWT in the CCT group were slightly higher than those in the CIT group at every test point, but the differences did not reach statistical significance (Tukey: TWL: *p* = 0.226, MWT: *p* = 0.051).

We also normalized the values of TWL and MWT (% of control) and observed the significant analgesia effect during and after tDCS in CIT and CCT groups (Figures 4B,D).

### Intensity-Dependent Antinociceptive Effects of Repetitive tDCS in CCI Rats

After confirming the location of stimulation electrode in the above studies, we chose different stimulation intensities and simulation times to determine the most effective stimulation current.

**Figure 5:**
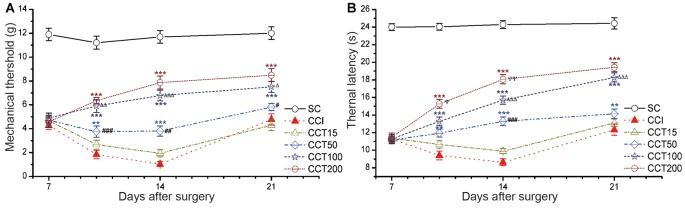
Effects of repetitive tDCS on mechanical allodynia and thermal hyperalgesia with different stimulation intensities. **(A)** MWT. **(B)** TWL. Five sessions daily of con-tDCS (20 min) with different intensity administrations started on day 7 after CCI surgery. Sham CCI (SC); CCI + 15 μA con-tDCS treatment (CCT15); CCI + 50 μA con-tDCS treatmen (CCT50); CCI + 100 μA con-tDCS treatment (CCT100); CCI + 200 μA con-tDCS treatment (CCT200). All behavior tests were examined at days 7, 10, 14, and 21 after CCI surgery. Statistical significance was analyzed by two-way ANOVA followed by Tukey’s *post hoc* test. **p < 0.01, ***p < 0.001 vs. CCI group; ^ #^*p* < 0.05, ^##^*p* < 0.01, ^###^*p* < 0.001 vs. CCT15 group; ^Δ^*p* < 0.05, ^ΔΔ^*p* < 0.01, ^ΔΔΔ^*p* < 0.001 vs. CCT50 group; ^ψ^*p* < 0.05, ^ψψ^*p* < 0.01 vs. CCT100 group.

Increasing stimulation current intensity resulted in an intensity-dependent increase in TWL of the ipsilateral hind paw after five repetitive A-tDCS (two-way repeated measures ANOVA: TWL: *F*_(5,60)_ = 336.733, *p* = 0.000; MWT: *F*_(5,60)_ = 172.656, *p* = 0.000; Figure 5). Compared to the CCI group, the CCT50, CCT100 and CCT200 induced significant increases in TWL and MWT during and after stimulation (Tukey’s test of groups: TWL and MWT: all *p* = 0.000), but the CCT15 did not induced analgesia effect (TWL: CCT15 *p* = 0.320; MWT: *p* = 0.833), The analgesic effects were higher in high intensity groups as compared to low intensity groups (Tukey’s test of groups: TWL: CCT50 vs. CCT15 *p* = 0.009, CCT100 vs. CCT50 *p* = 0.000, CCT200 vs. CCT100 *p* = 0.009; MWT: CCT50 vs. CCT15 *p* = 0.018, CCT100 vs. CCT50 *p* = 0.000, CCT200 vs. CCT100 *p* = 0.589; Figure 5).

### Time-Dependent Antinociceptive Effects of Repetitive tDCS in CCI Rats

**Figure 6:**
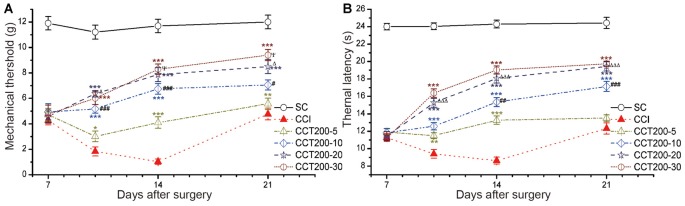
Effects of repetitive tDCS on mechanical allodynia and thermal hyperalgesia with different stimulation times. **(A)** MWT. **(B)** TWL. Five sessions daily of con-tDCS (200 μA) with different time administrations started on day 7 after CCI surgery. Sham CCI (SC); CCI + 5 min con-tDCS treatment (CCT200-5); CCI + 10 min con-tDCS treatment (CCT200-10); CCI + 20 min con-tDCS treatment (CCT200-20); CCI + 30 min con-tDCS treatment (CCT200-30). All behavior tests were examined at days 7, 10, 14 and 21 after CCI surgery. Statistical significance was analyzed by two-way ANOVA followed by Tukey’s *post hoc* test. **p < 0.01, ***p < 0.001 vs. CCI group; ^#^*p* < 0.05, ^##^*p* < 0.01, ^###^*p* < 0.001 vs. CCT200-5 group; ^Δ^*p* < 0.05, ^ΔΔΔ^*p* < 0.001 vs. CCT200-10 group; ^ψ^*p* < 0.05 vs. CCT200-20 group.

We observed time-dependent increases in pain thresholds in the ligated hind paw after five daily 200 μA con-tDCS, time points used were 5 min, 10 min, 20 min and 30 min (two-way repeated measures ANOVA: TWL: *F*_(5,60)_ = 347.503, *p* = 0.000; MWT: *F*_(5,60)_ = 132.278, *p* = 0.000; Figure 6). The CCT200-5, CCT200-10, CCT200-20 and CCT200-30 groups increased the pain values sharply during the test periods (Tukey’s test of groups: TWL and MWT: all *p* = 0.000). We also observed time-dependent increases in pain thresholds (Tukey’s test of groups: TWL: CCT200-5 vs. CCT200-10 *p* = 0.000, CCT200-10 vs. CCT200-20 *p* = 0.000, CCT200-20 vs. CCT200-30 *p* = 0.681; MWT: CCT200-5 vs. CCT200-10 *p* = 0.000, CCT200-10 vs. CCT200-20 *p* = 0.030, CCT200-20 vs. CCT200-30 *p* = 0.967).

### Proper Intervention Time Enhanced the Long-Term Antinociceptive Effects of Repetitive tDCS in CCI Rats

**Figure 7:**
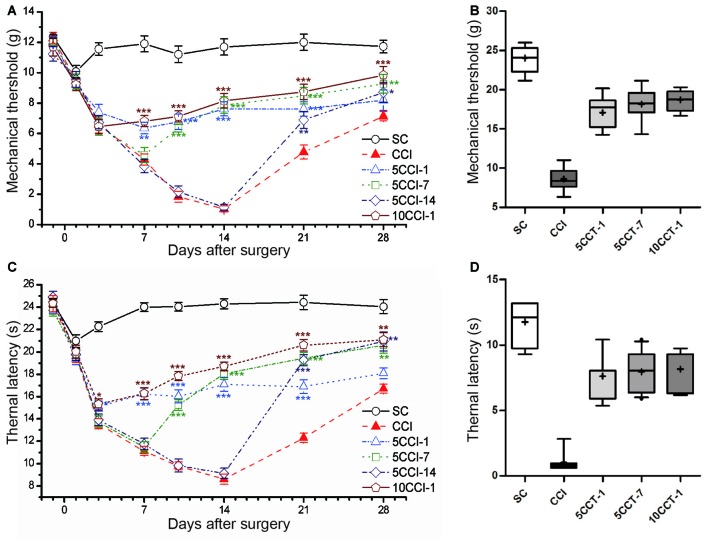
Effects of repetitive tDCS on mechanical allodynia and thermal hyperalgesia with different intervention times. **(A)** MWT. **(B)** Boxplot graph of MWT at day 14 after CCI. **(C)** TWL. **(D)** Boxplot graph of TWL at day 14 after CCI. Five or 10 sessions repetitive tDCS (20 min and 200 μA) with different intervention times were administered started on days 1, 7 and 14 following CCI surgery, respectively. Sham CCI (SC); CCI plus five sessions daily con-tDCS from day 1 after CCI (5CCT-1); CCI plus five sessions daily con-tDCS from day 7 after CCI (5CCT-7); CCI plus five sessions daily con-tDCS from day 14 after CCI (5CCT-14); CCI plus 10 sessions con-tDCS from day 1 after CCI (10CCT-1). All behavior tests were tested 1 day before the CCI surgery and at days 1, 3, 7, 10, 14, 21 and 28 after CCI surgery. Statistical significance was analyzed by two-way ANOVA followed by Tukey’s *post hoc* test. *p < 0.05, **p < 0.01, ***p < 0.001 vs. CCI group.

Intervention time also played a role in the antinociceptive effects following repetitive tDCS, the after-effects were different depending on the time of intervention of tDCS (two-way repeated measures ANOVA: TWL: *F*_(5,60)_ = 258.796, *p* = 0.000; MWT: *F*_(5,60)_ = 160.171, *p* = 0.000; Figures 7A,C).

When intervention was performed at 1 day after CCI (5CCT-1), repetitive A-tDCS maintained pain thresholds of the MWT and the TWL in the days following tDCS (Tukey’s test of groups: all: *p* = 0.000 vs. CCI). Given tDCS 7 days after CCI (5CCT-7) reversed the development of pain thresholds which gradually approached normal (Tukey’s test of groups: all: *p* = 0.000 vs. CCI). Pain thresholds were began to recover 14 days after CCI, and giving A-tDCS at this time point (5CCT-14) greatly reduced the recovery time and increased pain thresholds (Tukey’s test of groups: TWL: *p* = 0.000, MWT: *p* = 0.639 vs. CCI). We also observed using twice the number of stimulation sessions (10CCT-1) was helpful in reducing mechanical allodynia and thermal hyperalgesia (Tukey’s test of groups: all: *p* = 0.000 vs. CCI). The antinociceptive effects of 10CCT-1 was greater than those observed in the 5CCT-7 group following thermal hyperalgesia (TWL: *p* = 0.000: MWT: *p* = 0.379 vs. 5CCT-7; Figures 7A,C). Moreover, we found that inter-quartile range in the 10CCT-1 group was more centralized than that for other groups on 14th day following CCI, which reflected the spread of the threshold data (one-way measures ANOVA: TWL: *F*_(4,50)_ = 122.89, *p* = 0.000; MWT: *F*_(4,50)_ = 75.96, *p* = 0.000; Figures 7B,D).

### Antinociceptive Effects in Female Rats after Repetitive tDCS

**Figure 8:**
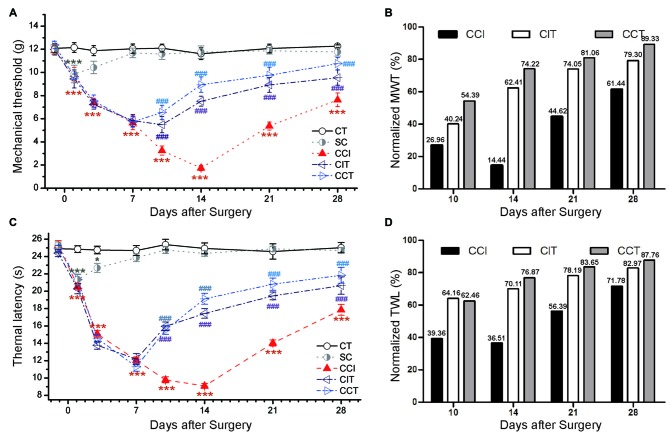
Effects of repetitive tDCS on mechanical allodynia and thermal hyperalgesia with different locations in female rats. **(A)** MWT. **(B)** Normalized of MWT. **(C)** TWL. **(D)** Normalized of TWL. Five sessions daily of tDCS (200 μA, 20 min) were administration started on day 7 following CCI surgery. CT (control); SC (sham CCI); CIT (CCI + ip-tDCS treatment); CCT (CCI + con-tDCS treatment). CIT and CCT treatments significantly increased the values of TWL and MWT. All behavior tests were tested 1 day before the CCI surgery and on days 1, 3, 7, 10, 14, 21 and 28 after CCI surgery. Statistical significance was analyzed by two-way ANOVA followed by Tukey’s *post hoc* test. *p < 0.05, ***p < 0.001 vs. CT group, ^###^*p* < 0.001 vs. CCI group.

A similar pain tend was observed in female rats compared to male rats following CCI, the TWL and MWT were significantly decreased in CCI rats compared to CT rats (Tukey’s test of groups: TWL and MWT: all *p* = 0.000; Figure 8). Consecutive sessions of tDCS (both CIT and CCT) induced antinociceptive effects which lasted for at least 1 week after stimulation in female CCI rats (two-way repeated measures ANOVA: TWL: *F*_(4,50)_ = 248.424, *p* = 0.000; MWT: *F*_(4,50)_ = 136.015, *p* = 0.000). Compared to the CCI group, A-tDCS significantly increased the pain values to a high level in both CIT group (Tukey’s test of groups: TWL and MWT: all *p* = 0.000) and CCT group (Tukey’s test of groups: TWL and MWT: all *p* = 0.000). We also observed that the mechanical allodynia and thermal hyperalgesia of the CCT group were mildly increased compared to the CIT group, but the differences did not reach statistical significance (Tukey’s test of groups: TWL: *p* = 0.074; MWT: *p* = 0.093; Figure 8). We also normalized the values of TWL and MWT (% of control) and observed the significant analgesia effect during and after tDCS (Figures 8B,D).

## Discussion

Clinical treatment of neuropathic pain is still a major challenge because of its long duration and difficulty in managing (Brunoni et al., 2012). As a non-invasive electrical stimulation treatment, tDCS technology has been used for many years in clinical settings (Gandiga et al., 2006; Brunoni et al., 2012). Radiological and clinical practice have proven that tDCS can play a role in the plasticity of CNS regulation and serve as a treatment for neuronal abnormalities (Borckardt et al., 2012; Brunoni et al., 2012; DosSantos et al., 2014; Woods et al., 2016). However, as systematical basic research is lacking, clinical effects are inconsistent and critical stimulus parameters are uncertain (Lee et al., 2015; Dedoncker et al., 2016). In this article, we choose a rat CCI model as the neuropathic pain model, which is the most common pain model that could finely simulate the clinical chronic neuropathic pain as reported by previous studies (Xiao et al., 2010; Ou et al., 2011; He et al., 2012). The pain threshold of MWT and TWL were evaluated at the different times after CCI.

Previous clinical studies chose the M1 cerebral cortex as the stimulus location because of its roles in the modulation of chronic pain, as it receives pain-related information from the thalamus and the somatosensory cortex (Nguyen et al., 2011; Mylius et al., 2012). Earlier findings indicated that the neural activity of contralateral M1 to an injured paw was increased after CCI (Ooi et al., 2006), and contralateral M1 stimulation has been used as a clinical treatment of chronic pain with the use of transdural motor cortex stimulation (MCS) and rTMS (Lefaucheur, 2006; Fontaine et al., 2009; Young et al., 2014). However, previous work has also reported that M1 stimulation with MCS or rTMS ipsilateral to injury could also significantly suppress pain-related responses in rats and human (Nahmias et al., 2009; Lucas et al., 2011; Viisanen et al., 2012), but there have been no extensive experimental study testing tDCS. In our study, the anode stimulation electrode was mounted contralateral or ipsilateral to the injured paw to measure the antinociceptive effect of tDCS, and our results demonstrated that both locations of A-tDCS led to significant decreases in pain. Our study supplements previous experiments in which the location of the electrode was not taken into consideration (Cioato et al., 2016; Filho et al., 2016). A possible mechanism of these results is supra-spinal antinociceptive activities via multiple parallel pathways (Pertovaara and Wei, 2003). Recent studies had found that M1 stimulation can also active the adjacent regions, including the periaqueductal gray, anterior cingulate cortex and amygdale (Nguyen et al., 2011). Further experiments have shown that rTMS enhances the corticospinal inhibitory system which might in turn mediate M1 stimulation-induced spinal antinociception (Rojas-Piloni et al., 2010; Dall Agnol et al., 2014). The transduction mechanism of antinociception release needs further study in the future.

We observed that the effects of A-tDCS on reducing hyperalgesia and allodynia depended on stimulus intensity and time. Stronger intensities or longer duration correlates with more charged input to the cortex (Nitsche and Paulus, 2011), and a better after-effect on relieving pain (Nitsche et al., 2008). In Series 2, we found the analgesic effect of single tDCS was diminished following 2 h, but repetitive tDCS prolongs the duration of analgesic effect (Series 3). We found that greater charge inputs given 1 week after original stimulation maintained high pain thresholds when re-assessed on days 21 and 28 following CCI. Therefore, to create more effective and long-lasting after-effects, we should increase the stimulation duration and the current intensity. In the last few decades, there has been consensus that increasing stimulation duration prolongs the occurrence and duration of after-effects in humans and animals (Nitsche et al., 2008), which we verified in our study. However, increasing of duration and intensity has limitations. Considering the safety of tDCS, we choose 200 μA as the highest intensity because in our preliminary experiments we observed transient tremors if the intensity was over 220 μA. On the other hand, we found that there were no statistical differences in pain thresholds between CCT200-30 and CCT200-20 groups (Figure 6), suggesting that the antinociceptive effects of tDCS may be saturated after 20 min of tDCS at 200 μA. As such, it is necessary to determine the best intervention time if the stimulation intensity and duration cannot be further increased to continue prolonging the after-effects of tDCS.

CCI was first reported by Bennett and Xie (1988) and was regarded as a typical model in neuropathic pain research. The duration of neuropathic pain was divided into two parts (Figure 2). Part one was described as the “progression period” and referred to days 1 through 14 following CCI, peaking between days 10 and 14; part two was described as “recovery period” and referred to the period following day 14 in which the sensitivity to pain decreased daily. We chose two time points (1 and 7 days after surgery) in the progression period and one point in the recovery period (14 days after surgery) for delivering tDCS. There were substantial signs of recovery in each treatment group compared to the CCI group: (1) repetitive A-tDCS reversed the decreased thresholds observed in CCI rats at every time point; (2) early intervention prevented the sharp decrease in the pain threshold, but did not restore the threshold to its normal level; (3) as chronic pain worsened, the degree of antinociception following tDCS decreased and the after-effect were not well maintained; (4) antinociceptive effects were present when tDCS was delivered throughout the progressed period; and (5) pain thresholds significantly improved when tDCS were given at the beginning of the recovery period (Figure 7).

To the best of our knowledge, we are the first to discover that intervention time is another key factor that influences the efficacy of stimulation. With the same intensity and duration, the efficacy of analgesia was prolonged and consolidated by choosing the right time points when stimulation is participated in the pain processing and extending the original session of tDCS.

Clinically, A-tDCS is widely used in chronic pain treatment, an intensity of 2 mA and duration of 20 min with five or seven daily repetitions are usually chosen, parameters that have proven effects (Mori et al., 2010; Ngernyam, 2014). However, other researchers have also been obtained in which A-tDCS was ineffective in the treatment of chronic pain using the same conditions (Ihle et al., 2014; Nardone et al., 2014) The patient described in this study had stable chronic pain for at least 6 months with a high VAS scores before the stimulation, however, intervention time was not considered during therapy. A proper intervention time, the number of session and the length of stimulus duration should be considered and may be dependent on different states and causes of illness (Ihle et al., 2014). Our results suggest that clinicians should consider personalized treatment in patients with chronic neuropathic pain, pay attention to specific stimulus parameters and disease characteristics.

Furthermore, brain lesions were reported when the current density was greater than 142.9 A/m^2^ in rat experiments. Our studies used a maximum current density of 63.69 A/m^2^ (200 μA/3.14 mm^2^) which is not associated with any tissue injure after tDCS (Liebetanz et al., 2009).

Futhermore, we examined the effect of tDCS in female rats. Previous work found gender-related differences in utilitarian behavior after tDCS with greater effects in females as compared to males (Chaieb et al., 2008). For CCI rats, the chronic nociceptive processing was similar in both sexes, but male rats responded more quickly than females to a thermal nociceptive stimulus, and the stimulus elicited less robust thermal hyperalgesic symptoms in males than in females (Tall et al., 2001). In our study, we also found hyperalgesia and allodynia in female CCI rats. Both ip- and con-tDCS had the similar antinociceptive effects in female CCI rats. However, the hormonal fluctuate could be interfere in the nociceptive response (Tall et al., 2001) and the mechanism of gender difference needs further study in the future.

There is increasing evidence that the after-effects of tDCS are not only driven by the regulation of inhibition and excitation, but also by the modification of synapses (Nguyen et al., 2011; Stagg and Nitsche, 2011). Another view is that tDCS does not only elicit that rapid depolarization required to produce action potentials in neurons, but also may produces long-lasting changes in cortical excitability and activity (Mylius et al., 2012). Consistent with this, another key hypothesis holds that chronic pain is likely to employ highly selective synaptic connections and molecular signaling pathways within pain-related cortical areas (Zhuo, 2008, 2013), resulting in cortical plasticity in both the descending and ascending systems. In the rat with peripheral injured, bilateral M1 receives pain-related information from the thalamus and the somatosensory cortex that maps to the injured paw (Xie et al., 2009; Ossipov et al., 2010). Stimulation of M1 might induce plasticity changes and reorganizations in the expression of neurotransmitter receptors (Lefaucheur et al., 2010; Stagg and Nitsche, 2011) which might include tonic activation of NMDA receptors (Pertovaara and Wei, 2003; Nguyen et al., 2011) and an enhanced anti-hypersensitivity effect in dopamine receptors (Viisanen et al., 2012). We observed increased NMDA receptors in bilateral M1 regions in CCI rats after repetitive tDCS (unpublished results). Therefore, the changes in NMDA receptors after tDCS might decrease the function of brain areas related to pain management through long-term potentiation (LTP) synaptic efficacy, thereby inducing cortical reorganization and CNS network processing. These affects are likely to reintroduce an optimal excitation/inhibition balance that allows for optimal homeostatic plasticity (Nitsche and Paulus, 2011; Stagg and Nitsche, 2011; Krause et al., 2013; Ngernyam, 2014).

The present study demonstrates the antinociceptive effect of tDCS in the male and female CCI rats. Both ip-DCS and con-tDCS produce a long-lasting analgesic effect on neuropathic pain, and the optimal stimulation parameters of tDCS are future studied. These dates may be helpful for the clinical applications of tDCS in pain control. More investigations on the synaptic mechanisms of tDCS should be conducted in the future.

## Author Contributions

H-ZR and H-ZW conceived and designed the study. H-ZW wrote the manuscript. S-HG, Y-DZ and W-JH carried out the animals’ experiments. H-ZW and X-LT participated in the treatment of tDCS. H-ZW and S-HG participated in data analyses and arranged the figures. All authors have read and approved the final manuscript.

## Conflict of Interest Statement

The authors declare that the research was conducted in the absence of any commercial or financial relationships that could be construed as a potential conflict of interest.

## References

[B1] Bennett G. J. Xie Y. K. (1988). A peripheral mononeuropathy in rat that produces disorders of pain sensation like those seen in man. Pain 33, 87–107. 10.1016/0304-3959(88)90209-6 2837713

[B2] Bindman L. J. Lippold O. C. J. Redfearn J. W. (1964). The action of brief polarizing currents on the cerebral cortex of the rat (1) during current flow and (2) in the production of long-lasting after-effects. J. Physiol. 172, 369–382. 10.1113/jphysiol.1964.sp007425 14199369 PMC1368854

[B3] Borckardt J. J. Bikson M. Frohman H. Reeves S. T. Datta A. Bansal V.. (2012). A pilot study of the tolerability and effects of high-definition transcranial direct current stimulation (HD-tDCS) on pain perception. J. Pain 13, 112–120. 10.1016/j.jpain.2011.07.001 22104190

[B4] Brunoni A. R. Nitsche M. A. Bolognini N. Bikson M. Wagner T. Merabet L.. (2012). Clinical research with transcranial direct current stimulation (tDCS): challenges and future directions. Brain Stimul. 5, 175–195. 10.1016/j.brs.2011.03.002 22037126 PMC3270156

[B5] Chaieb L. Antal A. Paulus W. (2008). Gender-specific modulation of short-term neuroplasticity in the visual cortex induced by transcranial direct current stimulation. Vis. Neurosci. 25, 77–81. 10.1017/s0952523808080097 18282312

[B6] Chaplan S. R. Bach F. W. Pogrel J. W. Chung J. M. Yaksh T. L. (1994). Quantitative assessment of tactile allodynia in the rat paw. J. Neurosci. Methods 53, 55–63. 10.1016/0165-0270(94)90144-9 7990513

[B7] Cioato S. G. Medeiros L. F. Marques Filho P. R. Vercelino R. de Souza A. Scarabelot V. L.. (2016). Long-lasting effect of transcranial direct current stimulation in the reversal of hyperalgesia and cytokine alterations induced by the neuropathic pain model. Brain Stimul. 9, 209–217. 10.1016/j.brs.2015.12.001 26775175

[B8] Dall Agnol L. Medeiros L. F. Torres I. L. S. Deitos A. Brietzke A. Laste G.. (2014). Repetitive transcranial magnetic stimulation increases the corticospinal inhibition and the brain-derived neurotrophic factor in chronic myofascial pain syndrome: an explanatory double-blinded, randomized, sham-controlled trial. J. Pain 15, 845–855. a 10.1016/j.jpain.2014.05.001 24865417

[B9] Dedoncker J. Brunoni A. R. Baeken C. Vanderhasselt M. (2016). A Systematic review and meta-analysis of the effects of transcranial direct current stimulation (tDCS) over the dorsolateral prefrontal cortex in healthy and neuropsychiatric samples: influence of stimulation parameters. Brain Stimul. 9, 501–517. 10.1016/j.brs.2016.04.006 27160468

[B10] DosSantos M. F. Martikainen I. K. Nascimento T. D. Love T. M. DeBoer M. D. Schambra H. M.. (2014). Building up analgesia in humans via the endogenous mu-opioid system by combining placebo and active tDCS: a preliminary report. PLoS One 9:e102350. 10.1371/journal.pone.0102350 25029273 PMC4100885

[B11] Dworkin R. H. O’Connor A. B. Kent J. Mackey S. C. Raja S. N. Stacey B. R.. (2013). Interventional management of neuropathic pain: NeuPSIG recommendations. Pain 154, 2249–2261. 10.1016/j.pain.2013.06.004 23748119 PMC4484720

[B12] Ferrucci R. Cortese F. Priori A. (2015). Cerebellar tDCS: how to do it. Cerebellum 14, 27–30. 10.1007/s12311-014-0599-7 25231432 PMC4318979

[B13] Filho P. R. M. Vercelino R. Cioato S. G. Medeiros L. F. de Oliveira C. Scarabelot V. L.. (2016). Transcranial direct current stimulation (tDCS) reverts behavioral alterations and brainstem BDNF level increase induced by neuropathic pain model: long-lasting effect. Prog. Neuropsychopharmacol. Biol. Psychiatry 64, 44–51. 10.1016/j.pnpbp.2015.06.016 26160698

[B14] Fontaine D. Hamani C. Lozano A. (2009). Efficacy and safety of motor cortex stimulation for chronic neuropathic pain: critical review of the literature. J. Neurosurg. 110, 251–256. 10.3171/2008.6.17602 18991496

[B15] Fregni F. Boggio P. S. Lima M. C. Ferreira M. J. L. Wagner T. Rigonatti S. P.. (2006). A sham-controlled, phase II trial of transcranial direct current stimulation for the treatment of central pain in traumatic spinal cord injury. Pain 122, 197–209. 10.1016/j.pain.2006.02.023 16564618

[B16] Gandiga P. C. Hummel F. C. Cohen L. G. (2006). Transcranial DC stimulation (tDCS): a tool for double-blind sham-controlled clinical studies in brain stimulation. Clin. Neurophysiol. 117, 845–850. 10.1016/j.clinph.2005.12.003 16427357

[B17] Hargreaves K. Dubner R. Brown F. Flores C. Joris J. (1988). A new and sensitive method for measuring thermal nociception in cutaneous hyperalgesia. Pain 32, 77–88. 10.1016/0304-3959(88)90026-7 3340425

[B18] He W. Cui J. Du L. Zhao Y. Burnstock G. Zhou H.. (2012). Spinal P2X7 receptor mediates microglia activation-induced neuropathic pain in the sciatic nerve injury rat model. Behav. Brain Res. 226, 163–170. 10.1016/j.bbr.2011.09.015 21933684

[B19] Ihle K. Rodriguez-Raecke R. Luedtke K. May A. (2014). tDCS modulates cortical nociceptive processing but has little to no impact on pain perception. Pain 155, 2080–2087. 10.1016/j.pain.2014.07.018 25083928

[B20] Krause B. Márquez-Ruiz J. Cohen Kadosh R. C. (2013). The effect of transcranial direct current stimulation: a role for cortical excitation/inhibition balance? Front. Hum. Neurosci. 7:602. 10.3389/fnhum.2013.00602 24068995 PMC3781319

[B21] Laste G. Caumo W. Adachi L. N. S. Rozisky J. R. de Macedo I. C. Filho P. R. M.. (2012). After-effects of consecutive sessions of transcranial direct current stimulation (tDCS) in a rat model of chronic inflammation. Exp. Brain Res. 221, 75–83. 10.1007/s00221-012-3149-x 22752510

[B22] Lee M. Kim Y. H. Im C. H. Kim J. H. Park C. H. Chang W. H.. (2015). What is the optimal anodal electrode position for inducing corticomotor excitability changes in transcranial direct current stimulation? Neurosci. Lett. 584, 347–350. 10.1016/j.neulet.2014.10.052 25450146

[B23] Lefaucheur J. Holsheimer J. Goujon C. Keravel Y. Nguyen J. (2010). Descending volleys generated by efficacious epidural motor cortex stimulation in patients with chronic neuropathic pain. Exp. Neurol. 223, 609–614. 10.1016/j.expneurol.2010.02.008 20188091

[B24] Lefaucheur J. P. (2006). The use of repetitive transcranial magnetic stimulation (rTMS) in chronic neuropathic pain. Neurophysiol. Clin. 36, 117–124. 10.1016/j.neucli.2006.08.002 17046606

[B25] Liebetanz D. Fregni F. Monte-Silva K. K. Oliveira M. B. Amâncio-dos-Santos A. Nitsche M. A.. (2006). After-effects of transcranial direct current stimulation (tDCS) on cortical spreading depression. Neurosci. Lett. 398, 85–90. 10.1016/j.neulet.2005.12.058 16448754

[B26] Liebetanz D. Koch R. Mayenfels S. König F. Paulus W. Nitsche M. A. (2009). Safety limits of cathodal transcranial direct current stimulation in rats. Clin. Neurophysiol. 120, 1161–1167. 10.1016/j.clinph.2009.01.022 19403329

[B27] Lucas J. M. Ji Y. Masri R. (2011). Motor cortex stimulation reduces hyperalgesia in an animal model of central pain. Pain 152, 1398–1407. 10.1016/j.pain.2011.02.025 21396776 PMC3098950

[B28] Mori F. Codecà C. Kusayanagi H. Monteleone F. Buttari F. Fiore S.. (2010). Effects of anodal transcranial direct current stimulation on chronic neuropathic pain in patients with multiple sclerosis. J. Pain 11, 436–442. 10.1016/j.jpain.2009.08.011 20018567

[B29] Mylius V. Borckardt J. J. Lefaucheur J. (2012). Noninvasive cortical modulation of experimental pain. Pain 153, 1350–1363. 10.1016/j.pain.2012.04.009 22633979

[B30] Nahmias F. Debes C. de Andrade D. C. Mhalla A. Bouhassira D. (2009). Diffuse analgesic effects of unilateral repetitive transcranial magnetic stimulation (rTMS) in healthy volunteers. Pain 147, 224–232. 10.1016/j.pain.2009.09.016 19822394

[B31] Nardone R. Höller Y. Leis S. Höller P. Thon N. Thomschewski A.. (2014). Invasive and non-invasive brain stimulation for treatment of neuropathic pain in patients with spinal cord injury: a review. J. Spinal Cord Med. 37, 19–31. 10.1179/2045772313Y.0000000140 24090372 PMC4066547

[B32] Ngernyam N. (2014). Transcranial direct current stimulation in neuropathic pain. J. Pain Relief s3:1 10.4172/2167-0846.1000s3-001 PMC4193292 25309825

[B33] Nguyen J. P. Nizard J. Keravel Y. Lefaucheur J. P. (2011). Invasive brain stimulation for the treatment of neuropathic pain. Nat. Rev. Neurol. 7, 699–709. 10.1038/nrneurol.2011.138 21931348

[B35] Nitsche M. A. Cohen L. G. Wassermann E. M. Priori A. Lang N. Antal A.. (2008). Transcranial direct current stimulation: state of the art 2008. Brain Stimul. 1, 206–223. 10.1016/j.brs.2008.06.004 20633386

[B34] Nitsche M. A. Paulus W. (2011). Transcranial direct current stimulation—update 2011. Restor. Neurol. Neurosci. 29, 463–492. 10.3233/RNN-2011-0618 22085959

[B37] O’Connell N. E. Wand B. M. Marston L. Spencer S. Desouza L. H. (2011). Non-invasive brain stimulation techniques for chronic pain. A report of a Cochrane systematic review and meta-analysis. Eur. J. Phys. Rehabil. Med. 47, 309–326. 21494222

[B36] O’Neill F. Sacco P. Nurmikko T. (2015). Evaluation of a home-based transcranial direct current stimulation (tDCS) treatment device for chronic pain: study protocol for a randomised controlled trial. Trials 16:186. 10.1186/s13063-015-0710-5 25902771 PMC4411773

[B38] Ooi Y. Satomura Y. Seki J. Yanagida T. Seiyama A. (2006). Optical coherence tomography reveals *in vivo* cortical plasticity of adult mice in response to peripheral neuropathic pain. Neurosci. Lett. 397, 35–39. 10.1016/j.neulet.2005.12.010 16386846

[B39] Ossipov M. H. Dussor G. O. Porreca F. (2010). Central modulation of pain. J. Clin. Invest. 120, 3779–3787. 10.1172/JCI43766 21041960 PMC2964993

[B40] Ou S. Zhao Y. Xiao Z. Wen H. Cui J. Ruan H. (2011). Effect of lappaconitine on neuropathic pain mediated by P2X3 receptor in rat dorsal root ganglion. Neurochem. Int. 58, 564–573. 10.1016/j.neuint.2011.01.016 21272608

[B41] Pertovaara A. Wei H. (2003). A dissociative change in the efficacy of supraspinal versus spinal morphine in the neuropathic rat. Pain 101, 237–250. 10.1016/s0304-3959(02)00320-2 12583866

[B42] Roizenblatt S. Fregni F. Gimenez R. Wetzel T. Rigonatti S. P. Tufik S.. (2007). Site-specific effects of transcranial direct current stimulation on sleep and pain in fibromyalgia: a randomized, sham-controlled study. Pain Pract. 7, 297–306. 10.1111/j.1533-2500.2007.00152.x 17986164

[B43] Rojas-Piloni G. Martínez-Lorenzana G. Condés-Lara M. Rodríguez-Jiménez J. (2010). Direct sensorimotor corticospinal modulation of dorsal horn neuronal C-fiber responses in the rat. Brain Res. 1351, 104–114. 10.1016/j.brainres.2010.06.010 20542015

[B44] Soler M. D. Kumru H. Pelayo R. Vidal J. Tormos J. M. Fregni F.. (2010). Effectiveness of transcranial direct current stimulation and visual illusion on neuropathic pain in spinal cord injury. Brain 133, 2565–2577. 10.1093/brain/awq184 20685806 PMC2929331

[B45] Spezia Adachi L. N. Caumo W. Laste G. Fernandes Medeiros L. Ripoll Rozisky J. de Souza A.. (2012). Reversal of chronic stress-induced pain by transcranial direct current stimulation (tDCS) in an animal model. Brain Res. 1489, 17–26. 10.1016/j.brainres.2012.10.009 23063889

[B46] Stagg C. J. Nitsche M. A. (2011). Physiological basis of transcranial direct current stimulation. Neuroscientist 17, 37–53. 10.1177/1073858410386614 21343407

[B47] Tall J. M. Stuesse S. L. Cruce W. L. R. Crisp T. (2001). Gender and the behavioral manifestations of neuropathic pain. Pharmacol. Biochem. Behav. 68, 99–104. 10.1016/s0091-3057(00)00461-5 11274714

[B49] Viisanen H. Ansah O. B. Pertovaara A. (2012). The role of the dopamine D2 receptor in descending control of pain induced by motor cortex stimulation in the neuropathic rat. Brain Res. Bull. 89, 133–143. 10.1016/j.brainresbull.2012.08.002 22902996

[B50] Volz M. S. Volz T. S. Brunoni A. R. de Oliveira J. P. V. T. Fregni F. (2012). Analgesic effects of noninvasive brain stimulation in rodent animal models: a systematic review of translational findings. Neuromodulation 15, 283–295. 10.1111/j.1525-1403.2012.00478.x 22759345 PMC4018630

[B51] Woods A. J. Antal A. Bikson M. Boggio P. S. Brunoni A. R. Celnik P.. (2016). A technical guide to tDCS, and related non-invasive brain stimulation tools. Clin. Neurophysiol. 127, 1031–1048. 10.1016/j.clinph.2015.11.012 26652115 PMC4747791

[B52] Xiao Z. Ou S. He W. Zhao Y. Liu X. Ruan H. (2010). Role of midbrain periaqueductal gray P2X3 receptors in electroacupuncture-mediated endogenous pain modulatory systems. Brain Res. 1330, 31–44. 10.1016/j.brainres.2010.03.030 20302849

[B48] Xie Y.-F. Huo F.-Q. Tang J.-S. (2009). Cerebral cortex modulation of pain. Acta Pharmacol. Sin. 30, 31–41. 10.1038/aps.2008.14 19079295 PMC4006538

[B53] Young N. A. Sharma M. Deogaonkar M. (2014). Transcranial magnetic stimulation for chronic pain. Neurosurg. Clin. N. Am. 25, 819–832. 10.1016/j.nec.2014.07.007 25240669

[B54] Yu X. Li Y. Wen H. Zhang Y. Tian X. (2015). Intensity-dependent effects of repetitive anodal transcranial direct current stimulation on learning and memory in a rat model of Alzheimer’s disease. Neurobiol. Learn. Mem. 123, 168–178. 10.1016/j.nlm.2015.06.003 26070657

[B55] Zaghi S. Heine N. Fregni F. (2009). Brain stimulation for the treatment of pain: a review of costs, clinical effects, and mechanisms of treatment for three different central neuromodulatory approaches. J. Pain Manag. 2, 339–352. 20585474 PMC2888303

[B56] Zhuo M. (2008). Cortical excitation and chronic pain. Trends Neurosci. 31, 199–207. 10.1016/j.tins.2008.01.003 18329111

[B57] Zhuo M. (2013). Long-term potentiation in the anterior cingulate cortex and chronic pain. Philos. Trans. R. Soc. Lond. B Biol. Sci. 369:20130146. 10.1098/rstb.2013.0146 24298148 PMC3843878

[B58] Zimmermann M. (1983). Ethical guidelines for investigations of experimental pain in conscious animals. Pain 16, 109–110. 10.1016/0304-3959(83)90201-4 6877845

