# Supplementary material for: Parameter Optimization Analysis of Prolonged Analgesia Effect of tDCS on Neuropathic Pain Rats
Source: Front Behav Neurosci. 2017 Jun 13;11:115. doi: 10.3389/fnbeh.2017.00115 (PMC5468406; doi:10.3389/fnbeh.2017.00115)
Supplement: Supplementary file 1 [file Table_1.doc]

**Table 1**

Series with different tDCS parameters. F/M=Female/Male; stimulation location of anodal electrode was fixed onto the ipsilateral or contralateral M1 to the ligated hind paw; 1/7/14d means tDCS started on 1^st^/7^th^/14^th^ days after CCI injury, respectively; 1 session means single tDCS, and 5 sessions means 5 times daily tDCS, and 10 sessions means 10 times tDCS at two weeks; sham tDCS means the stimulation electrode was placed at the same positions as for real stimulation only with duration of 10 s. PT= pain thresholds, “-” means no operation.

| **Series** | **Group** | **Detail** | **Location** | **Intensity** | **Time** | **Session** | **Intervention time** | **F/M** |
| --- | --- | --- | --- | --- | --- | --- | --- | --- |
| 1. Changes of PT after CCI | CT | Control | - | - | - | - | - | M |
|  | SC | Sham CCI | - | - | - | - | - | M |
|  | CCI | CCI | - | - | - | - | - | M |
| 2.  Changes of PT after single A-tDCS on CCI rats | SC | Sham CCI | - | - | - | - | - | M |
|  | SCSIT | Shan CCI+ sham 200μA ip-tDCS | ipsilateral | 200μA | 10s | 1 | 14d | M |
|  | SCSCT | Shan CCI+ sham 200μA con-tDCS | contralateral | 200μA | 10s | 1 | 14d | M |
|  | SCIT | Sham CCI+  200μA ip-tDCS | ipsilateral | 200μA | 10s | 1 | 14d | M |
|  | SCCT | Sham CCI+  200μA con-tDCS | contralateral | 200μA | 10s | 1 | 14d | M |
|  | CCI | CCI | - | - | - | - | - | M |
|  | CSIT | CCI+ sham  200μA ip-tDCS | ipsilateral | 200μA | 10s | 1 | 14d | M |
|  | CSCT | CCI+ sham  200μA con-tDCS | contralateral | 200μA | 10s | 1 | 14d | M |
|  | CIT100 | CCI+ 100 μA ip-tDCS | ipsilateral | 100μA | 20min | 1 | 14d | M |
|  | CIT200 | CCI+200 μA ip-tDCS | ipsilateral | 200μA | 20min | 1 | 14d | M |
|  | CCT100 | CCI+100 μA con-tDCS | contralateral | 100μA | 20min | 1 | 14d | M |
|  | CCT200 | CCI+ 200 μA con-tDCS | contralateral | 200μA | 20min | 1 | 14d | M |
| 3  Changes of PT after repetitive tDCS with different locations | SC | Sham CCI | - | - | - | - | - | M |
|  | CCI | CCI | - | - | - | - | - | M |
|  | CIT | CCI+200 μA ip-tDCS | ipsilateral | 200μA | 20min | 5 | 7d | M |
|  | CCT | CCI+ 200 μA con-tDCS | contralateral | 200μA | 20min | 5 | 7d | M |
| 4 Changes of PT after repetitive con tDCS with different intensities | SC | Sham CCI | - | - | - | - | - | M |
|  | CCI | CCI | - | - | - | - | - | M |
|  | CCT15 | CCI+ 15 μA con-tDCS | contralateral | 15μA | 20min | 5 | 7d | M |
|  | CCT50 | CCI+ 50 μA con-tDCS | contralateral | 50μA | 20min | 5 | 7d | M |
|  | CCT100 | CCI+100 μA con-tDCS | contralateral | 100μA | 20min | 5 | 7d | M |
|  | CCT200 | CCI+ 200 μA con-tDCS | contralateral | 200μA | 20min | 5 | 7d | M |
| 5 Changes of PT after repetitive con tDCS with different times | SC | Sham CCI | - | - | - | - | - | M |
|  | CCI | CCI | - | - | - | - | - | M |
|  | CCT200-5 | CCI + 5 min con-tDCS | contralateral | 200μA | 5min | 5 | 7d | M |
|  | CCT200-10 | CCI + 10 min con-tDCS | contralateral | 200μA | 10min | 5 | 7d | M |
|  | CCT200-20 | CCI + 20 min con-tDCS | contralateral | 200μA | 20min | 5 | 7d | M |
|  | CCT200-30 | CCI + 30 min con-tDCS | contralateral | 200μA | 30min | 5 | 7d | M |
| 6 Changes of PT after repetitive con tDCS with different intervention times | SC | Sham CCI | - | - | - | - | - | M |
|  | CCI | CCI | - | - |  | - | - | M |
|  | 5CCT-1 | CCI+ tDCS | contralateral | 200μA | 20min | 5 | 1d | M |
|  | 5CCT-7 | CCI+ tDCS | contralateral | 200μA | 20min | 5 | 7d | M |
|  | 5CCT-14 | CCI+ tDCS | contralateral | 200μA | 20min | 5 | 14d | M |
|  | 10CCT-1 | CCI+ tDCS | contralateral | 200μA | 20min | 10 | 1d | M |
| 7  Changes of PT after repetitive tDCS on female CCI rats | CT | Control | - | - | - | - | - | F |
|  | SC | Sham CCI | - | - | - | - | - | F |
|  | CCI | CCI | - | - | - | - | - | F |
|  | CIT | CCI+200 μA ip-tDCS | ipsilateral | 200μA | 20min | 5 | 7d | F |
|  | CCT | CCI+ 200 μA con-tDCS | contralateral | 200μA | 20min | 5 | 7d | F |
